# Questionnaire Analysis on Incidence and Risk Factors of Dry Eye in Children From a Myopia Outpatient Clinic

**DOI:** 10.3389/fmed.2022.846709

**Published:** 2022-02-14

**Authors:** Ning Wang, XiaoYun Zhuang, XiaoWei Zhong, Ju Zhang, GuangWei Li, Suxia Li

**Affiliations:** ^1^School of Ophthalmology, Shandong First Medical University and Shandong Academy of Medical Sciences, Jinan, China; ^2^Department of Ophthalmology, Eye Hospital of Shandong First Medical University, Jinan, China; ^3^Department of Ophthalmology, School of Clinical Medicine, Weifang Medical University, Weifang, China; ^4^State Key Laboratory Cultivation Base, Shandong Provincial Key Laboratory of Ophthalmology, Shandong Eye Institute, Jinan, China

**Keywords:** dry eye, children, questionnaire, myopia outpatient, incidence and risk factors

## Abstract

**Objective:**

To analyze the incidence and risk factors of dry eye in children from a myopia outpatient clinic *via* a questionnaire and Keratograph 5M.

**Methods:**

A cross–sectional study was performed. sThere were 214 children (428 eyes) selected from the myopia outpatient clinic of the affiliated Eye Hospital of Shandong First Medical University from July 2021 to September 2021, including 105 boys (210 eyes) and 109 girls (218 eyes), with an average age of 10.1 ± 2.5 years. The incidence rate and influence factors for dry eye were calculated.

**Results:**

Thirty–four of 214 children were diagnosed with dry eye, accounting for 15.9% of the patients. The correlation between fussy eating and the tear meniscus height was statistically significant (*Z* = −2.158, *p* = 0.039), along with the correlation between short–distance use of eyes and the tear meniscus height (*Z* = −2.135, *p* = 0.033). The degree of meibomian gland deficiency was graded. The meibomian gland was graded as grade 1 in 242 eyes (68.9%), grade 2 in 104 eyes (29.6%), and grade 3 in 5 eyes (1.4%). There was a significant difference in the correlation between eye rubbing and the incidence of dry eye in children (*Z* = −2.747, *p* = 0.008). There was also a significant difference in the correlation between picky eating and the incidence of dry eye in children (*Z* = −2.347; *p* = 0.024). There was a statistically significant correlation between the time of looking at electronic products and the morphology of the meibomian gland (*Z* = −2.201, *p* = 0.028). The results showed that the effect of mild and moderate ametropia on the non–invasive tear breakup time in children was statistically significant (*Z* = −2.027; *p* = 0.043).

**Conclusion:**

There is a high incidence of dry eye in children in the myopia outpatient clinic. There is a significant correlation between picky eating, eye rubbing, and the incidence of dry eye. Looking at electronic products for a long time will also affect the morphology of the meibomian gland in children.

## Introduction

Dry eye is a common chronic eye disease. In this condition, the noninvasive tear breakup time (NIBUT) is shortened because of the instability of the tear film of the patient. The cornea is exposed to the external environment, and the tear evaporates too fast, causing a series of discomforts, such as burning and dryness in the eyes (1). It is reported that the incidence of dry eye has reached 21–30% in Chinese adults, with varying incidence rates in different regions (2, 3). However, the incidence rate of dry eye in children has not been reported in detail because of unsatisfactory cooperation among patients, poor expression, and lack of attention (4). Environmental pollution, the popularity of electronic products, and lifestyle changes in recent years have led to continuous increases in the incidence of dry eye in China. Moreover, the incidence rate of dry eye in children is increasing year by year; indeed, dry eye has become the most common ophthalmic disease other than ametropia among adults (5, 6). In this study, dry eye in 218 children (436 eyes) from the myopia outpatient clinic at Shandong First Medical University Eye Hospital was investigated using questionnaires and a Keratograph 5M. The incidence rate of dry eye in children and the risk factors for dry eye in children were analyzed.

## Methods

### Research Objective

In the study, 214 children (428 eyes) were selected from the myopia outpatient clinic of the Eye Hospital of Shandong First Medical University from July 2021 to August 2021, including 105 boys (210 eyes) and 109 girls (218 eyes). The age distribution was 4–17 years, with an average of 10.1 ± 2.5 years.

The inclusion criteria were as follows: (1) Participants had no active ocular organic diseases, (2) Participants were able to complete the questionnaire independently or accompanied by their guardians, and (3) Participants had no systemic diseases or systemic diseases involving the eyes. The exclusion criteria were as follows: (1) history of eye surgery; and (2) The best corrected visual acuity was <0.8.

The trial was approved by the medical ethics committee of the affiliated ophthalmic hospital of Shandong First Medical University (approval: SDSYKYY202107) and complied with the Declaration of Helsinki. All the children and their guardians gave an informed consent and signed an informed consent form.

### Questionnaires and Examination Items

To exclude the active eye diseases, all participants underwent a detailed medical history collection, routine refractive examination, mydriasis free fundus photography, and slit lamp microscopy in the myopia outpatient clinic. All subjects and their guardians fulfilled the 5–item Dry Eye Questionnaire (DEQ−5) and the Children's Dry Eye Questionnaire that is developed for this study under the guidance of professionals. The NIBUT, tear meniscus height (TMH), and meibomian gland morphology were determined using the Keratograph 5M.

The questions on the Children's Dry Eye Questionnaire were as follows:

(1) Are there dry eye symptoms, including dryness, burning, fatigue, heaviness, redness, photophobia, itching, eye pain, tears, and increased secretion?
(2) Does the patient wear contact lenses or orthokeratology lenses?
(3) Does the patient blink frequently (>20 blinks/min or abnormal blinks, such as winking and frowning)?
(4) Has the frequency of eye rubbing increased abnormally recently?
(5) Is there is a history of allergic rhinitis?
(6) Is there any history of eyelid conjunctiva, such as wheat granuloma, chalazion, entropion, trichiasis, blepharitis, or conjunctivitis?
(7) Is there a history of eye trauma?
(8) Does the patient often look at electronic products?
(9) What types of electronic products often used (e.g., mobile phones, tablets, computers, televisions, ink screens, or other electronic products)?
(10) How much time is spent looking at electronic products every day (<1 h, 1–3 h, more than 3 h)?
(11) much time does the patient spend using his/her eyes for close work every day, such as reading paper books and doing homework (<1 h, 1–3 h, more than 3 h)?
(12) Does the patient prefer certain foods (a lover of fried foods, sweets, meat, and too little fruit and vegetables)?
(13) If the patient prefer certain foods, what are the patient dietary preferences. (meat, grains, vegetables, fruits, fried foods, sweets)?
(14) Is the patient to the following substances: pollen, catkins, insects and mites, pets?
(15) How much time do you spend participating in outdoor activities every day (<1 h, 1–3 h, more than 3 h)?
(16) Is your reading posture correct?
(17) Are you exposed to secondhand smoke?

We adopted the DEQ−5 questionnaire, which is more suitable for children than the is Ocular Surface Disease Index (OSDI) (7). A Keratograph 5M was used to collect the dry eye data of all the subjects, including the NIBUT, the TMH, and any meibomian gland loss. All subjects were examined by the same examiner at the same room temperature. Meibomian gland photography was finally collected to avoid eye irritation, which could affect the data accuracy of NIBUT and TMH, caused by eyelid turnover.

### Dry Eye Diagnosis Index

The diagnostic criteria of patients with dry eye from the “Consensus of Chinese Dry Eye Experts: Examination and Diagnosis” (2020) were used. Specifically, the criteria are as follows: the child complains of one of the subjective symptoms, such as dry eye, foreign body sensation, burning, fatigue, discomfort, red eye, or vision fluctuation, or the score of the child on the DEQ−5 questionnaire is >6 points but NIBUT is <5 s.

Should a child fit these criteria, he/she is considered to have dry eye.

On the other hand, the degree of meibomian gland deletion can be divided into four grades, as follows: grade 1, in which the degree of meibomian gland deletion is 0–25%, or there is no deletion but the meibomian gland has different degrees of distortion; grade 2, in which the degree of deletion is 26–50%; grade 3, in which the degree of meibomian gland deficiency is 51–75%; and grade 4, in which the degree of deletion is 76–100%.

### Statistical Methods

The IBM SPSS Statistics 26.0 statistical software was used for the analysis. The measurement data were tested for K–S normality, and the measurement data with non–normal distribution were presented as median (25%, 75%). The Mann–Whitney *U*-test was used to analyze the difference of different groups. A *p* < 0.05 was considered statistically significant. The following values were assigned to the content of the questionnaire: gender: male = 1, female = 2; duration of viewing electronic products: <1 h = 3, 1–3 h = 2, more than 3 h = 1; exposure to secondhand smoke: yes = 1, no = 2; history of eyelid conjunctiva: yes = 1, no = 2; rubbing eyes frequently: yes = 1, no = 2; picky eater: yes = 1, no = 2; correct reading posture: yes = 1, no = 2; frequent blinking: yes = 1, no = 2; and history of allergy: yes = 1, no = 2.

## Results

**Figure 1:**
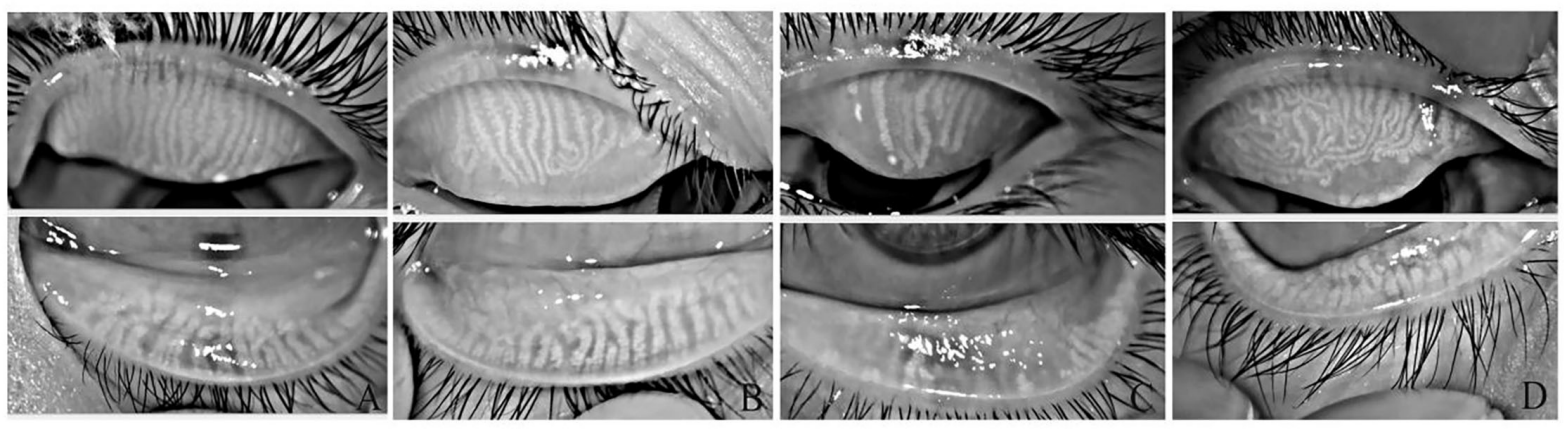
Different meibomian gland morphologies in ametropic children. **(A)** 0–25% area of loss. **(B)** 25–50% area of loss. **(C)** 50–75% area of loss. **(D)** Meibomian glands were twisted, and some meibomian glands were twisted more than 90°.

Among the 214 patients, 34 were diagnosed with dry eye, accounting for 15.9% of the sample. Conversely, there were 180 patients without dry eye, accounting for 84.1% of the sample. There were 351 meibomian gland images collected in 428 eyes, accounting for 82% of the sample. Meibomian gland deletion was graded using Image J software. The degree of meibomian gland deletion was grade 1 (no deletion) in 242 eyes (68.9%), grade 2 (mild deletion) in 104 eyes (29.6%), and grade 3 (severe deletion) in 5 eyes (1.4%; Figure 1).

**Figure 2:**
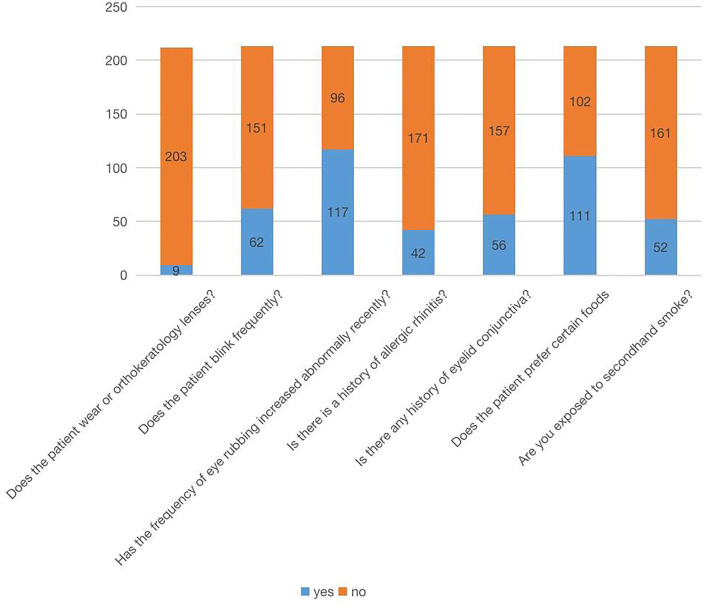
Questionnaire content and results.

**Figure 3:**
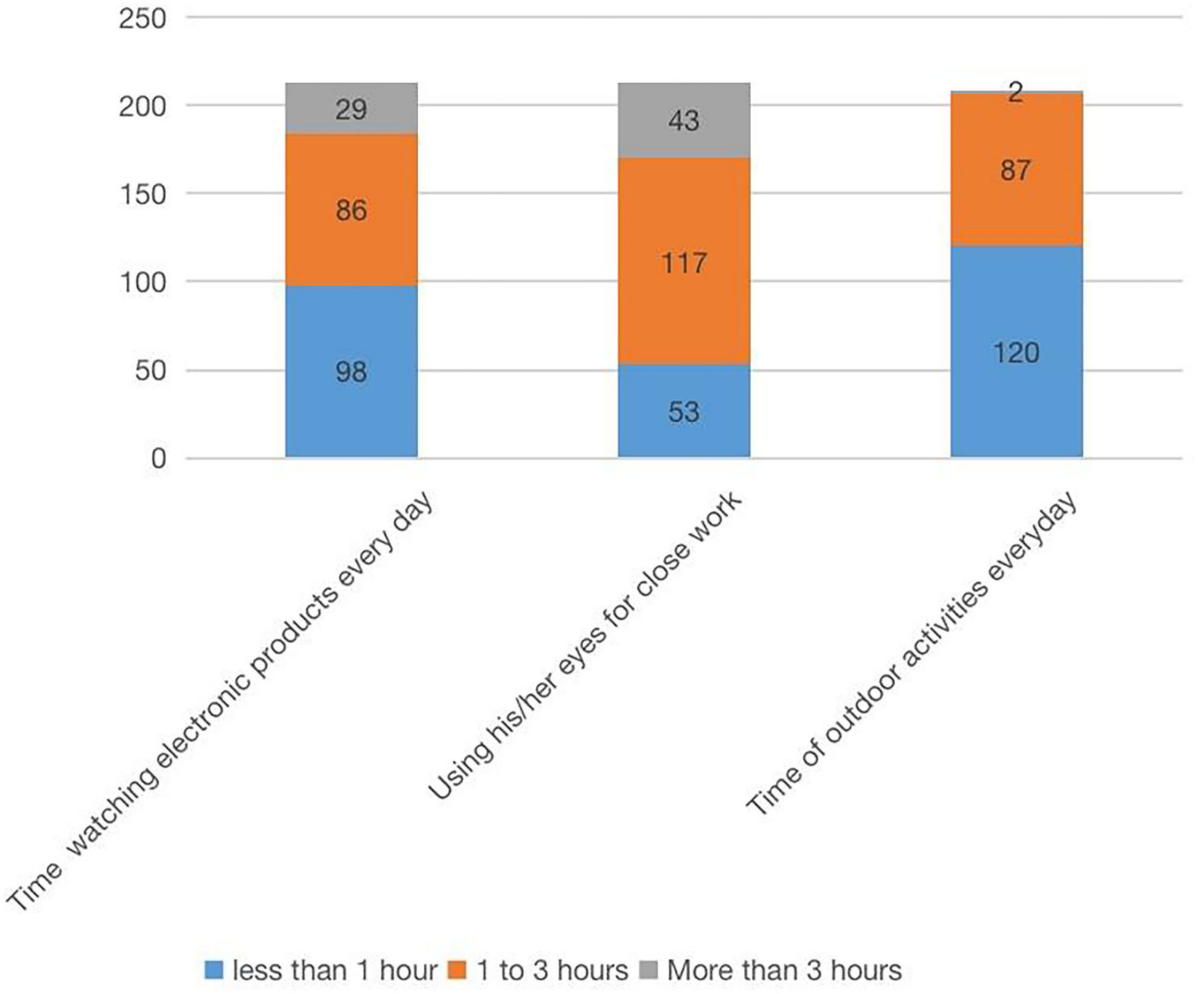
Questionnaire content and results.

**Figure 4:**
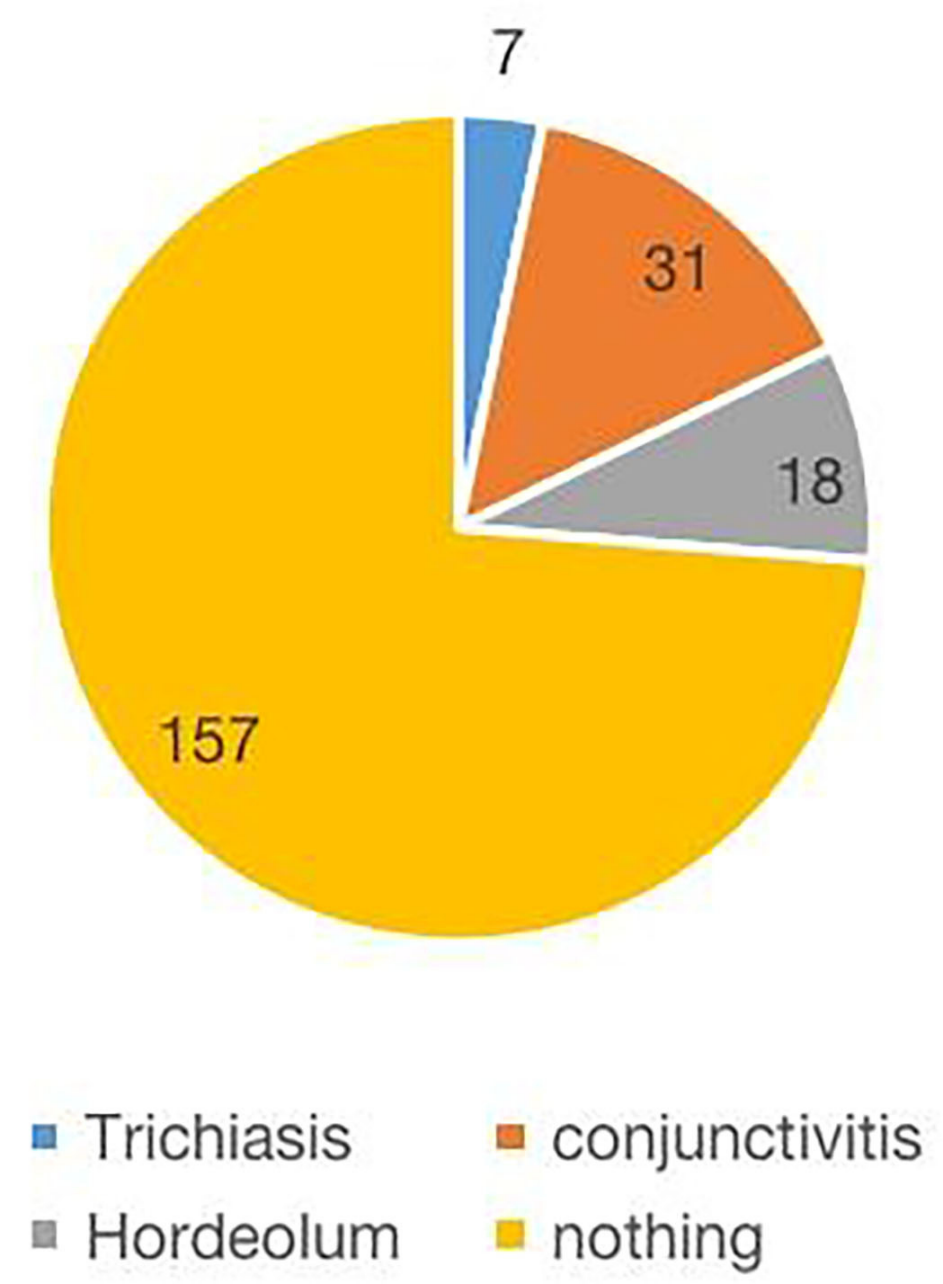
History distribution of eyelid conjunctiva.

A total of 213 questionnaires were collected. The results showed that 9 patients (4%) wore orthokeratology lenses, whereas 204 patients (96%) did not wear orthokeratology lenses. There were 62 patients (29.1%) with recent frequent blinking symptoms and 151 (70.9%) without such symptoms. There were 117 patients (54.9%) who often rubbed their eyes and 96 (45.1%) who did not. Forty–two patients (19.7%) had a history of allergy, whereas 171 (80.3%) had no history of allergy. There were 56 patients (26.3%) with palpebral conjunctiva history and 157 patients (73.7%) without palpebral conjunctiva history. Furthermore, 111 patients (52.1%) were picky and partial eaters, whereas 102 people (47.9%) were not picky and partial eaters; among them, the picky eaters preferred meat, fried food, and sweets. Incorrect reading posture was evident in 68 patients (31.9%) and correct reading posture in 145 patients (68.1%). Fifty–two patients (24.4%) were exposed to secondhand smoke, whereas 161 (75.6%) were not (Figures 2–4).

Data on a total of 428 eyes were collected, including 109 eyes with NIBUT ≤ 5 s (25.5%) and 319 eyes with NIBUT > 5 s (74.5%). The NIBUT ≤ 5 s was set to 1 and NIBUT > 5 s was set to 2. Data on TMH were collected for a total of 425 eyes collected, of which the TMH of 236 eyes was ≤0.2 mm (55.5%) and the TMH of 189 eyes was >0.2 mm (44.5%). If the TMH was <0.2 mm, the value was set to 1; if the TMH was >0.2 mm, the value was set to 2. The results showed that there was no statistical difference in the correlation between eye rubbing, fussy eating, exposure to secondhand smoke, history of eyelid conjunctiva, frequent blinking, history of allergy, time spent on electronics, short distance use of eyes, outdoor activity time, and the NIBUT (*Z* = −0.375, *p* = 0.738; *Z* = 1.051, *p* = 0.317; *Z* = 0.423, *p* = 0.698; *Z* = 0.353, *p* = 0.798; *Z* = 0.628, *p* = 0.541; *Z* = 1.201, *p* = 0.264; *Z* = 0.251, *p* = 0.802; *Z* = 1.457, *p* = 0.145; *Z* = 1.457, *p* = 0.145; *Z* = −0.032, *p* = 0.974; Table 1). The correlation between fussy eating and the TMH was statistically significant (*Z* = −2.158, *p* = 0.039), and the correlation between short distance use of eyes and the TMH was statistically significant (*Z* = −2.135, *p* = 0.033). There was no statistical difference in the correlation between eye rubbing, exposure to secondhand smoke, history of eyelid conjunctiva, frequent blinking, history of allergy, time spent on electronics, outdoor activity time and the TMH (*Z* = −1.383, *p* = 0.168; *Z* = 0.127, *p* = 0.910; *Z* = 0.721, *p* = 0.498; *Z* = 1.066, *p* = 0.331; *Z* = 0.695, *p* = 0.537; *Z* = 1.308, *p* = 0.191; *Z* = −0.006, and *p* = 0.995; Table 2).

**Table 1:** Effects of different factors on the noninvasive tear breakup time (NIBUT).

**Influence factor**	**NIBUT <5 s**	**NIBUT > 5 s**	**Mann–Whitney** ***U*****-test**	
			***Z*–value**	***p*–value**
Eye rubbing	1.00 (1.00, 2.00)	1.00 (1.00, 2.00)	−0.375	0.738
Picky eating	1.00 (1.00, 2.00)	1.00 (1.00, 2.00)	−1.051	0.317
Exposure to secondhand smoke	2.00 (1.00, 2.00)	2.00 (2.00, 2.00)	−0.423	0.698
History of eyelid conjunctiva	1.00 (1.00, 1.00)	1.00 (1.00, 2.00)	−0.353	0.798
Frequent blinking	2.00 (1.00, 2.00)	2.00 (1.00, 2.00)	−0.628	0.541
Any history of allergy	2.00 (2.00, 2.00)	2.00 (2.00, 2.00)	−1.201	0.264
Looking at electronics	2.00 (2.00, 3.00)	2.00 (2.00, 3.00)	−0.251	0.802
Using his/her eyes for close work	2.00 (1.00, 2.00)	2.00 (2.00, 2.00)	−1.457	0.145
Outdoor activity time everyday	1.00 (1.00, 2.00)	1.00 (1.00, 2.00)	−0.032	0.974

**Table 2:** Effects of different factors on the tear meniscus height (TMH).

**Influence factor**	**TMH <0.2 mm**	**TMH > 0.2 mm**	**Mann–Whitney** ***U*****-test**	
			***Z*–value**	***p*–value**
Eye rubbing	1.00 (1.00, 2.00)	1.00 (1.00, 2.00)	−1.149	0.280
Picky eating	1.00 (1.00, 2.00)	2.00 (1.00, 2.00)	**–**2.438	0.019
Exposure to secondhand smoke	2.00 (1.00, 2.00)	2.00 (2.00, 2.00)	−0.590	0.572
History of eyelid conjunctiva	1.00 (1.00, 1.00)	1.00 (1.00, 2.00)	−1.014	0.367
Frequent blinking	2.00 (1.00, 2.00)	2.00 (1.00, 2.00)	−0.979	0.336
Any history of allergy	2.00 (2.00, 2.00)	2.00 (2.00, 2.00)	−1.067	0.327
Looking at electronics	2.00 (2.00, 3.00)	2.00 (2.00, 3.00)	−1.308	0.194
Using his/her eyes for close work	2.00 (1.00, 2.00)	2.00 (2.00, 3.00)	−2.135	0.035
Outdoor activity time everyday	1.00 (1.00, 2.00)	1.00 (1.00, 2.00)	−0.006	1.000

The influencing factors that may affect the dry eye disease of children were statistically analyzed using the Mann-Whitney *U*-test. The results showed that there was a significant difference in the correlation between eye rubbing and the incidence of dry eye in children (*Z* = −2.747, *p* = 0.008). Furthermore, there was a significant difference in the correlation between picky eating and the incidence of dry eye in children (*Z* = −2.347, *p* = 0.024). There was no significant correlation between age, gender, exposure to secondhand smoke, frequent blinking, history of allergy, time to see electronic products, using your eyes for close work every day, time spent on outdoor activities, and the incidence of dry eye in children (*Z* = −0.477, *p* = 0.636; *Z* = 1.374, *p* = 0.193; *Z* = −1.607, *p* = 0.128; *Z* = −1.275, *p* = 0.220; *Z* = −0.163, *p* = 1.000; *Z* = −0.153, *p* = 0.898; *Z* = −0.388, *p* = 0.748, *Z* = −0.463, and *p* = 0.674; Table 3).

**Table 3:** Influence of various factors on the prevalence of dry eye.

**Influence factor**	**Dry eye group**	**Undiagnosed dry eye group**	**Mann–Whitney** ***U*****-test**	
			***Z*–value**	***p*–value**
Age	10.00 (8.00, 12.00)	10.00 (8.00, 11.00)	−0.477	0.636
Gender	2.00 (1.00, 2.00)	1.00 (1.00, 2.00)	−1.374	0.193
Eye rubbing	1.00 (1.00, 1.25)	1.00 (1.00, 2.00)	−2.747	0.008
Picky eating	1.00 (1.00, 2.00)	2.00 (1.00, 2.00)	−2.347	0.024
Exposure to secondhand smoke	2.00 (1.00, 2.00)	2.00 (2.00, 2.00)	−1.607	0.128
Frequent blinking	2.00 (1.00, 2.00)	2.00 (1.00, 2.00)	−1.275	0.220
Any history of allergies	2.00 (2.00, 2.00)	2.00 (2.00, 2.00)	−0.163	1.000
Look at electronics	2.50 (2.00, 3.00)	2.00 (2.00, 3.00)	−0.153	0.898
Using his/her eyes for close work	2.00 (1.00, 2.00)	2.00 (2.00, 2.00)	−0.388	0.748
Outdoor activity time everyday	1.00 (1.00, 2.00)	1.00 (1.00, 2.00)	−0.463	0.674

Because of the poor cooperation of some subjects, which meant that meibomian gland images could not be taken, 350 meibomian gland images were collected in 428 eyes, accounting for 81.78% of all eyes. Among them, 126 eyes (36%) had normal meibomian glands, whereas 224 (64%) had meibomian gland loss or meibomian gland distortion. Data were assigned with the following values: normal meibomian gland = 0, and meibomian gland loss or meibomian gland distortion = 1 (meibomian gland distortion was defined as one or more gland distortions >90°; Figure 4). The influencing factors of meibomian gland loss were analyzed using the Mann–Whitney *U*-test. There was a statistically significant correlation between the time spent looking at electronic products and the morphology of the meibomian gland (*Z* = −2.201, *p* = 0.028). There was no significant correlation in terms of exposure to secondhand smoke (*Z* = −1.712, *p* = 0.090) and whether patients had a history of eyelid conjunctiva (*Z* = −1.542, *p* = 0.127), eye rubbing (*Z* = −0.787, *p* = 0.436), and meibomian gland morphology (Table 4).

**Table 4:** Analysis of influencing factors of meibomian gland deletion.

**Influence factor**	**Normal meibomian gland group**	**Meibomian gland deletion group**	**Mann–Whitney** ***U*****-test**	
			***Z*–value**	***p*–value**
Length of looking at electronic products	3.00 (2.00, 3.00)	2.00 (2.00, 3.00)	−2.201	0.028
Exposure to secondhand smoke	2.00 (2.00, 2.00)	2.00 (1.00, 2.00)	−1.712	0.090
Any history of eyelid conjunctiva	1.00 (1.00, 1.00)	1.00 (1.00, 2.00)	−1.542	0.127
Eye rubbing	1.00 (1.00, 2.00)	1.00 (1.00, 2.00)	−0.787	0.436

Data were collected for a total of 288 out of 428 eyes. The Best Corrected Visual Acuity (BCVA) of all eyes was >4.9, including mild ametropia where myopia and astigmatism were ±0.5D ~ ± 3D and moderate ametropia, where myopia and astigmatism were > ± 3D. Mild ametropia was assigned 1 and moderate ametropia was assigned 2. The effects of refractive status on NIBUT and the incidence of dry eye were analyzed using the Mann–Whitney *U*-test. The results showed that the effect of mild and moderate ametropia on the incidence of dry eyes in children was not statistically significant (*Z* = −0.795, *p* = 0.453), whereas the effect of mild and moderate ametropia on the NIBUT in children was statistically significant (*Z* = −2.027, *p* = 0.043) (Table 5).

**Table 5:** Effects of ametropia on tear film rupture time and dry eye incidence in children.

**Parameter**	**Mild ametropia**	**Moderate ametropia**	**Mann–Whitney** ***U*****-test**	
			***Z*–value**	***p*–value**
Dry eye diagnosed	2.00 (2.00, 2.00)	2.00 (2.00, 2.00)	−0.795	0.453
NIBUT(S)	9.18 (5.35, 14.91)	7.46 (3.54, 14.15)	−2.027	0.043

## Discussion

Dry eye is a chronic disease caused by the shortening of NIBUT because of the abnormal quality, quantity, and dynamics of tears (8). The latest epidemiology shows that nearly 400 million people in China are troubled by dry eye symptoms (9), but most of the previously researched groups comprised adults. In the past, it was believed that eye conditions of children were better than those of adults, and the incidence of dry eye disease in children was lower. When it did arise, it was thought to be mostly caused by congenital, endocrine, or other diseases, such as ectodermal hypoplasia, Sjogren syndrome, and so on (10). However, home quarantine measures during the Corona Virus Disease 2019 (COVID−19) epidemic, the increase in children's online education, and close eye use may lead to an increase in the incidence of dry eye and ametropia in children. This article discusses the missing condition of meibomian glands, the incidence rate of dry eye in children, and the risk factors of dry eye in children who are eye outpatients in Shandong Province to improve the understanding of doctors on dry eye.

It has been reported that the incidence of dry eye in children worldwide is 0.2–36% (11–14). The incidence of dry eye in the 214 children included in this study was 15.9%, and the results were similar to those in previous studies. We found a high proportion of children with dry eyes who often rubbed their eyes and were picky eaters. Balasubramanian et al. (15) showed that protease activity and levels of inflammatory factor matrix metalloproteinase 9 (MMP−9), interleukin 6 (IL−6), and tumor necrosis factor α (TNF–α) significantly increased after eye rubbing. Recent studies (16) have shown that dry eye is a chronic inflammatory response. Inflammatory factors destroy the connecting barrier between corneal cells, resulting in damage to corneal epithelial cells. The abscission of the corneal epithelium intensifies the inflammatory reaction process, finally leading to a vicious circle. Soifer et al. (17) found that the increase of MMP−9 may be related to a reduced tear secretion. However, there is no evidence that regular eye rubbing causes the occurrence of dry eye, and extensive research is still needed on the relationship between eye rubbing and dry eye.

Our questionnaire shows that children who are picky and partial eaters prefer meat, fried foods, and sweets, which may lead to an insufficient intake of various trace elements. Studies have shown that vitamin A deficiency may be related to the occurrence of dry eye. Vitamin A deficiency will lead to the drying of the cornea and conjunctiva, damage to conjunctival goblet cells, and the reduction of mucin secretion, which will affect the quality of tear film, resulting in the occurrence of dry eye symptoms (18). Research findings showed that the dry eye symptoms of a Ω-3 fatty acid group were significantly less than those of a placebo group after 12 months (19). Picky eating can also lead to obesity, and Ω-3 fatty acids can delay the progress of obesity (20, 21). The research of Gupta et al. (22) showed that meibomian gland curvature is significantly associated with higher body mass index (BMI) in children, and meibomian gland morphological abnormality is considered to be a sensitive indicator of meibomian gland dysfunction (23). The research of Bu et al. (24) showed that a high fat diet can also cause meibomian gland inflammation in mice. Therefore, picky eating may reduce the intake of vitamin A, Ω-3 fatty acids, and other trace elements. The lack of trace elements affects the morphology of the meibomian gland, the quality of tear film, and the TMH, leading to the occurrence of dry eye.

We observed the meibomian gland photos of 351 eyes, which showed that in about 30% of the eyes, the meibomian gland atrophy was more than 25%. We speculate that meibomian gland atrophy may occur prior to dry eye symptoms, which may be due to the compensatory function of an increased tear secretion. Our analysis of the factors that may lead to the loss of meibomian glands in children shows that the longer we watch electronic products, the more severe the atrophy of the meibomian gland becomes. Research shows that looking at electronic products for a long time will lead to a decrease in blinking frequency and an increase in incomplete blinking times. The discharge of meibum mainly occurs through the mechanical force of the eyelid covering the corneal surface in a blink. A complete blink is helpful to increase the thickness of the lipid layer on the eye surface, whereas an incomplete blink will reduce the lipid discharge and the thickness of the lipid layer on the eye surface (25, 26).

If a large amount of meibum is deposited in the meibomian gland, resulting in the expansion and bending of the gland, the gland will have a chronic inflammation. Finally, the meibomian gland will gradually shrink. Ning and Zhao (27) showed that meibomian gland atrophy was more serious in children exposed to video terminals for a long time, whereas the degree of dry eye and NIBUT in children exposed for a short time were correlated with incomplete blinking. In addition, we found that the increase in refractive power may affect the NIBUT, which may be due to the change in tear film morphology and visual fatigue caused by ametropia, resulting in uneven tear film distribution. Studies have shown that there is a significant correlation between ametropia and the incidence of dry eye (28–30).

Our study found that the incidence of dry eye in children in the myopia outpatients of our hospital was 15.9%. There was a significant correlation between eye rubbing, picky eating, and the incidence of dry eye in children. We speculate that this may be related to the increase of inflammatory factors after eye rubbing and the insufficient intake of Ω-3 fatty acids and vitamin A. We also found deletion of the meibomian gland in many children. Meibomian gland shrinking may be related to watching electronic products for a long time. In conclusion, the attention of doctors should be paid to dry eye in children because of the high incidence of dry eye and high degree of meibomian gland deletion in this population. This study also has some shortcomings, such as a small sample size.

## Data Availability Statement

The raw data supporting the conclusions of this article will be made available by the authors, without undue reservation.

## Ethics Statement

The studies involving human participants were reviewed and approved by Ethics Committee of Shandong Ophthalmic Hospital. Written informed consent to participate in this study was provided by the participants' legal guardian/next of kin. Written informed consent was obtained from the individual(s), and minor(s)' legal guardian/next of kin, for the publication of any potentially identifiable images or data included in this article.

## Author Contributions

NW: writing and revising papers and data statistics and analysis. XZhu, XZho, and JZ: data statistics and analysis and collecting data. GL: providing ideas, data statistics and analysis, and collecting data. SL: design topics, writing guidance, paper revision, and providing guidance. All authors contributed to the article and approved the submitted version.

## Funding

This study was supported by the Shandong Medical and Health Science and Technology Development Plan (202107020804).

## Conflict of Interest

The authors declare that the research was conducted in the absence of any commercial or financial relationships that could be construed as a potential conflict of interest.

## Publisher's Note

All claims expressed in this article are solely those of the authors and do not necessarily represent those of their affiliated organizations, or those of the publisher, the editors and the reviewers. Any product that may be evaluated in this article, or claim that may be made by its manufacturer, is not guaranteed or endorsed by the publisher.

## References

[1] Clayton JA. Dry Eye. N Engl J Med. (2018) 378:2212–23. 10.1056/NEJMra1407936 29874529

[2] Du J Liang QF. Risk factors of dry eye. Int Ophthalmol Overview. (2018) 42:189–93. 10.3760/cma.j.issn.1673-5803.2018.03.010

[3] Liu ZG. Emphasize on refinement and standardization of diagnosis and treatment of dry eye. Zhonghua yan ke za zhi. (2017) 53:641–4. 10.3760/cma.j.issn.0412-4081.2017.09.001 28926880

[4] Liu YL Wu HP. Research progress on related factors of dry eye in children. Guoji Yanke Zazhi. (2018) 18:1982–5. 10.3980/j.issn.1672-5123.2018.11.08

[5] Ning YX Zhao SZ. Incidence and risk factors of dry eye in children. Guoji Yanke Zenglan. (2020) 44:117–20. 10.3760/cma.j.issn.1673-5803.2020.02.00834912822

[6] Liu ZG Li W. Pay attention to the treatment and diagnosis of dry eye in children. Zhonghua yan ke za zhi. (2018) 54:406–8. 10.3760/cma.j.issn.0412-4081.2018.06.002 29895114

[7] Chidi-Egboka NC Golebiowski B Lee SY Vi M Jalbert I. Dry eye symptoms in children: can we reliably measure them? Ophthalmic Physiol Opt. (2021) 41:105–15. 10.1111/opo.12762 33222234

[8] Consensus of Chinese Dry Eye Experts: Definition and Classification (2020). Zhonghua yan ke za zhi. (2020) 56:418–22. 10.3760/cma.j.cn112142-20200316-00190

[9] Song P Xia W Wang M Chang X Wang J Jin S. Variations of dry eye disease incidence by age, sex and geographic characteristics in China: a systematic review and meta–analysis. J Glob Health. (2018 D) 8:020503. 10.7189/jogh.08.020503 30206477 PMC6122008

[10] Alves M Dias AC Rocha EM. Dry eye in childhood: epidemiological and clinical aspects. Ocul Surf. (2008) 6:44–51. 10.1016/S1542-0124(12)70104-0 18264654

[11] Dana R Bradley JL Guerin A Pivneva I Stillman IÖ Evans AM. Estimated incidence and incidence of dry eye disease based on coding analysis of a large, all–age United States health care system. Am J Ophthalmol. (2019) 202:47–54. 10.1016/j.ajo.2019.01.026 30721689

[12] Uchino M Dogru M Uchino Y. Japan Ministry of Health study on incidence of dry eye disease among Japanese high school students. Am J Ophthalmol. (2008) 146:925–9.e2. 10.1016/j.ajo.2008.06.030 18723141

[13] Donthineni PR Kammari P Shanbhag SS Singh V Das AV Basu S. Incidence, demographics, types and risk factors of dry eye disease in India: electronic medical records driven big data analytics report I. Ocul Surf. (2019) 17:250–6. 10.1016/j.jtos.2019.02.007 30802671

[14] Guo MX Xiang DM Yan LF Zhou J Liu W Hu LX.. Analysis of risk factors of associated with dry eye among 3–12 years old children in Guangzhou. Zhongguo xieshi yu xiaoer yanke zazhi. (2016) 24:11–15. 10.3969/J.ISSN.1005-328X.2016.02.004

[15] Balasubramanian SA Pye DC Willcox MD. Effects of eye rubbing on the levels of protease, protease activity and cytokines in tears: relevance in keratoconus. Clin Exp Optom. (2013) 96:214–8. 10.1111/cxo.12038 23496656

[16] Barabino S Chen Y Chauhan S Dana R. Ocular surface immunity: homeostatic mechanisms and their disruption in dry eye disease. Prog Retin Eye Res. (2012) 31:271–85. 10.1016/j.preteyeres.2012.02.003 22426080 PMC3334398

[17] Soifer M Mousa HM Stinnett SS Galor A Perez VL. Matrix metalloproteinase9 positivity predicts long term decreased tear production. Ocul Surf. (2021) 19:270–74. 10.1016/j.jtos.2020.10.003 33098983

[18] Sommer A. Xerophthalmia and vitamin A status. Prog Retin Eye Res. (1998) 17:9–31. 10.1016/S1350-9462(97)00001-3 9537797

[19] Dry Eye Assessment and Management Study Research Group Asbell PA Maguire G Pistilli M Ying GS Szczotka-Flynn LB Hardten DR. n−3 Fatty acid supplementation for the treatment of dry eye disease. N Engl J Med. (2018) 378:1681–90. 10.1056/NEJMoa1709691 29652551 PMC5952353

[20] Del-Río-Navarro BE Miranda-Lora AL Huang F Hall-Mondragon MS Leija-Martínez JJ. Effect of supplementation with omega−3 fatty acids on hypertriglyceridemia in pediatric patients with obesity. J Pediatr Endocrinol Metab. (2019) 32:811–9. 10.1515/jpem-2018-0409 31271554

[21] Simopoulos AP. An increase in the omega−6/omega−3 fatty acid ratio increases the risk for obesity. Nutrients. (2016 M) 8:128. 10.3390/nu8030128 26950145 PMC4808858

[22] Gupta PK Venkateswaran N Heinke J Stinnett SS. Association of meibomian gland architecture and body mass index in a pediatric population. Ocul Surf. (2020) 18:657–62. 10.1016/j.jtos.2020.06.009 32707337

[23] Adil MY Xiao J Olafsson J Chen X Lagali NS Ræder S. Meibomian gland morphology is a sensitive early indicator of meibomian gland dysfunction. Am J Ophthalmol. (2019) 200:16–25. 10.1016/j.ajo.2018.12.006 30578784

[24] Bu J Zhang M Wu Y Jiang N Guo Y He X. High–fat diet induces inflammation of meibomian gland. Invest Ophthalmol Vis Sci. (2021) 62:13. 10.1167/iovs.62.10.13 34398199 PMC8374999

[25] Portello JK Rosenfield M Chu CA. Blink rate, incomplete blinks and computer vision syndrome. Optom Vis Sci. (2013) 90:482–7. 10.1097/OPX.0b013e31828f09a7 23538437

[26] Wan T Jin X Lin L Xu Y Zhao Y. Incomplete blinking may attribute to the development of meibomian gland dysfunction. Curr Eye Res. (2016) 41:179–85. 10.3109/02713683.2015.1007211 25835130

[27] Ning YX Zhao SZ. The feature of meibomian gland and tear film lipid layer in dry eye of children with long–term visual display terminal exposure. Zhonghua yan ke za zhi. (2019):201–5. 10.3760/cma.j.issn.2095-0160.2019.03.008

[28] Guo YY Li RX. Effect of refractive state on dry eye in children. Zhongguo shiyong yanke zazhi. (2017) 35:295–8. 10.3760/cma.j.issn.1006-4443.2017.03.016

[29] Ilhan N Ilhan O Ayhan Tuzcu E Daglioglu MC Coskun M Parlakfikirer N. Is there a relationship between pathologic myopia and dry eye syndrome? Cornea. (2014) 33:169–71. 10.1097/ICO.0000000000000033 24322801

[30] Fahmy RM Aldarwesh A. Correlation between dry eye and refractive error in Saudi young adults using noninvasive Keratograph 4. Indian J Ophthalmol. (2018) 66:653–56. 10.4103/ijo.IJO_1103_17 29676308 PMC5939156

